# A deep learning segmentation strategy that minimizes the amount of manually annotated images

**DOI:** 10.12688/f1000research.52026.2

**Published:** 2022-01-17

**Authors:** Thierry Pécot, Alexander Alekseyenko, Kristin Wallace

**Affiliations:** 1 Department of Biochemistry and Molecular Biology, Hollings Cancer Center, Medical University of South Carolina, Charleston, SC, 29407, USA; 2 Rennes 1 University, SFR Biosit (UMS 3480 - US 018), F-35000 Rennes, France; 3 Departments of Public Health Sciences and Oral Health Sciences, Biomedical Informatics Center, Medical University of South Carolina, Charleston, SC, 29407, USA; 4 Department of Public Health Sciences, Medical University of South Carolina, Charleston, SC, 29407, USA

**Keywords:** Deep learning, image annotation, semantic and instance segmentations, conditional GANs, nuclei segmentation

## Abstract

Deep learning has revolutionized the automatic processing of images. While deep convolutional neural networks have demonstrated astonishing segmentation results for many biological objects acquired with microscopy, this technology's good performance relies on large training datasets. In this paper, we present a strategy to minimize the amount of time spent in manually annotating images for segmentation. It involves using an efficient and open source annotation tool, the artificial increase of the training dataset with data augmentation, the creation of an artificial dataset with a conditional generative adversarial network and the combination of semantic and instance segmentations. We evaluate the impact of each of these approaches for the segmentation of nuclei in 2D widefield images of human precancerous polyp biopsies in order to define an optimal strategy.

## Introduction

Over the last decade, deep learning approaches have outperformed all existing methods for image segmentation ^1– 4^. Semantic segmentation, the estimation of a label at each pixel, and instance segmentation, the identification of individual objects, were successfully applied to spatially characterize biological entities in microscopic images ^5– 8^. However, these powerful approaches rely on large annotated datasets. While more and more datasets become publicly available ^9, 10^, annotated data for every combination of modalities, tissues and biological objects is far from completion. If the number of images to be segmented is low, a fully manual workflow might be the most time efficient option. Otherwise, procedures to efficiently build training datasets are required to use the full potential of deep learning-based segmentation at a single biological lab scale.

In this paper, we propose a strategy to minimize the amount of time dedicated to manually annotate images and investigate several approaches to maximize accuracy when only using one annotated image. We apply this strategy to segment nuclei stained with DAPI in widefield images of human colorectal adenomas ( *i.e.* precancerous polyps) as follows. First, we take advantage of existing training datasets ^11, 12^ and massive data augmentation to obtain a preliminary segmentation. We then use an open source annotation software ^12^ to manually correct this segmentation and consequently define the training dataset. Next, we simulate synthetic images using a conditional generative adversarial network (GAN) ^13^ to increase the size of the training dataset. Finally, we combine U-Net ^14, 15^, a semantic segmentation approach, and Mask R-CNN ^16^, an instance segmentation approach, to improve the nuclear segmentation accuracy.

## Methods

### Sample preparation

In this study, we used the Medical University of South Carolina (MUSC) pathology laboratory information system CoPath (Cerner Corporation, Kansas City, MO), to identify a convenience sample of colorectal adenomas excised from patients who underwent a sigmoidoscopy or colonoscopy with polypectomy between October 2012 and May 2016. For each patient, we obtained a formalin-fixed, paraffin-embedded (FFPE) tissue block and prepared one H&E and 5, 5-micron sections for immunofluorescence (IF) on FFPE tissue. Prior to the start of the IF procedures, all antibodies were optimized and reviewed by the study immunologist, the pathologist, the epidemiologist, and laboratory personnel to ensure agreement and proper staining. The MUSC Institutional Review Board has approved the research study (IRB # PRO-00007139).

### Image acquisition

DAPI was used for nuclear counterstaining. Stained slides were mounted with ProLong™ Gold Antifade Reagent (Cat. # P36934, ThermoFisher) and imaged using the Akoya Vectra® Polaris™ Automated Imaging system (Akoya Biosciences, Marlborough, MA). Whole slide scans were done at 20X magnification and regions of interest where chosen randomly.

### Deep learning code

U-Net, Mask R-CNN and pix2pix were coded in Python using the Python libraries numpy ^17^, tensorflow ^18^, keras ^19^, scipy ^20^ and scikit-image ^21^.

### Training dataset

The training dataset consisted of three 1868 × 1400 images manually annotated with Annotater ^12^. Only one image was used to train U-Net and Mask R-CNN as well as pix2pix (conditional GAN) for most of the study, in addition to publicly available datasets (image set BBBC039v1 available from the Broad Bioimage Benchmark Collection ^9^ and a mouse intestinal epithelium dataset ^12^). The two other images were added to the training dataset in the last section to be compared with the combination of results obtained with U-Net and Mask R-CNN (see Figure 3a).

### U-Net training

The annotated 1868 × 1400 image was divided into six 622 × 700 images for training: five of these images were included in the training dataset while the last one defined the validation dataset. As U-Net is a semantic segmentation approach, three classes were defined to allow separating nuclei as proposed in 22: inner nuclei, nuclei contours and background. To facilitate nuclei separation, the nuclei contours between touching cells were dilated ^22^. To limit over-fitting, the imaging field for images in the training dataset was set to 256 × 256 by randomly cropping the 622 × 700 input images. These cropped images were then normalized to obtain intensity values between 0 and 1. A root mean square prop was used to estimate the parameters of the deep convolutional neural network by minimizing a weighted cross entropy loss to handle class imbalance for 100 epochs without data augmentation and 25 epochs with data augmentation. The weights associated with each class were defined from the training dataset as their inverse proportion. A data augmentation to increase the training dataset by a factor of 100 was processed after normalization with the imgaug python library ^23^ and included flipping, rotation, pixel dropout, blurring, noise addition and contrast modifications. In Figure 2 and Figure 3, augmented simulated images were obtained by applying the same modifications with the imgaug python library to simulated images with pix2pix. When combining the annotated image for this study with simulated images and/or existing datasets, the number of augmented images was defined to be balanced between the different data.

### U-Net post-processing

An ImageJ macro ^24, 25^ was used to convert the three classes obtained with U-Net to individual nuclei. More specifically, individual nuclei were identified by thresholding the subtraction of the nuclei contours component from the inner nuclei component with a threshold equal to 0.35. A 3D Voronoi tessellation ^26^ was then applied to assign each pixel to a nucleus. The object component was defined as all pixels whose background component was inferior to 0.95. This object component was then multiplied by the Voronoi tessellation to obtain individual nuclei. The Voronoi tessellation implies that a 1-pixel width area between nuclei is not assigned to any nucleus. To address this problem, the location of these pixels is obtained by subtracting the binary thresholding of the individual nuclei from the object component. The individual nuclei are then dilated ^27^ and multiplied to this subtraction to be added to the individual nuclei. Finally, nuclei with less than 35 pixels were removed.

### Mask R-CNN

The annotated 1868 × 1400 image was divided into thirty-five 266 × 280 images for training: thirty of these images were included in the training dataset while the last five images defined the validation dataset. Version 2.1 of Mask R-CNN ^16^ was used in this study. The backbone network was defined as the Resnet-101 deep convolutional neural network ^28^. We used the code in 5 to define the only class in this study, i.e. the nuclei. A data augmentation to increase the training dataset by a factor of 100 was processed before normalization with the imgaug python library ^23^ and included resizing, cropping, flipping, rotation, shearing, pixel dropout, blurring, sharpness and brightness modifications, noise addition and contrast modifications. Transfer learning with fine-tuning from a network trained on the coco dataset ^29^ was also applied. In the first epoch, only the region proposal network, the classifier and mask heads were trained. The whole network was then trained for the next three epochs. In Figure 2 and Figure 3, augmented simulated images were obtained by applying the same modifications with the imgaug python library to simulated images with pix2pix. When combining the annotated image for this study with simulated images and/or existing datasets, the number of augmented images was defined to be balanced between the different data. The maximum image size used for processing Mask R-CNN was larger than 256 as resizing and cropping were applied for data augmentation and set to 512. This parameter was defined as 1024 when other existing datasets were included for training as magnification in these images is higher.

### Evaluation

One 1868 × 1400 and one 934 × 1400 manually annotated images were used for evaluation. As proposed in 11, we used the F1 score with respect to the Intersection over Union ( *IoU*) to evaluate the different nuclei segmentation approaches. More formally, let **O**_*GT*_ = { *O _GT_* ( *e*)} _*e*=1,..., *n*_ be the set of *n* ground truth nuclei and **O**_*E*_ = { *O _E_* ( *e*)} _*e*=1,..., *m*_ be the set of *m* estimated nuclei. The *IoU* defined between the truth nucleus *O _GT_* ( *e*_1_) and the estimated nucleus *O _E_* ( *e*_2_) was defined as:

$$IoU(O_{GT}(e_{1}),\;O_{E}(e_{2}))\;=\frac{O_{GT}(e_{1})\cap O_{E}(e_{2})}{O_{GT}(e_{1})\cup O_{E}(e_{2})}.$$

An *IoU*( *O _GT_* ( *e*_1_), *O _E_* ( *e*_2_)) equal to 0 implies that *O _GT_* ( *e*_1_) and *O _E_* ( *e*_2_) do not share any pixel while an *IoU*( *O _GT_* ( *e*_1_), *O _E_* ( *e*_2_)) equal to 1 means that *O _GT_* ( *e*_1_) and *O _E_* ( *e*_2_) are identical. To ensure that one ground truth nucleus is not associated to multiple estimated nuclei and conversely, we use the following definition for the IoU:

$$IoU*(O_{GT}(e_{1}),\;O_{E}(e_{2}))\;=\left\{ \begin{array}{l} \frac{O_{GT}(e_{1})\cap O_{E}(e_{2})}{O_{GT}(e_{1})\cup O_{E}(e_{2})}\;\text{if}\;\begin{array}{c} \frac{O_{GT}(e_{1})\cap O_{E}(e_{2})}{O_{GT}(e_{1})\cup O_{E}(e_{2})}\;>\;\frac{O_{GT}(e_{1})\cap O_{E}(e_{i})}{O_{GT}(e_{1})\cup O_{E}(e_{i})}\;\forall i\in 1,\ldots ,m,\\ \frac{O_{GT}(e_{1})\cap O_{E}(e_{2})}{O_{GT}(e_{1})\cup O_{E}(e_{2})}>\;\frac{O_{GT}(e_{j})\cap O_{E}(e_{2})}{O_{GT}(e_{j})\cup O_{E}(e_{2})}\;\forall j\in 1,\ldots ,n, \end{array}\\ 0\;\text{otherwise.} \end{array}\right.$$

F1 score for a given IoU* threshold *t* > 0 can be defined as:

$$F1(t)\;=\frac{2\times TP(t)}{2\times TP(t)+FN(t)+FP\left(t\right)},$$

where

$$\begin{array}{c} TP(t)\;=\;\sum_{\begin{array}{l} e_{1}\in \{1,\ldots ,n\}\\ e_{2}\in \{1,\ldots ,m\} \end{array}}\;(IoU*(O_{GT}(e_{1}),\;O_{E}(e_{2}))>t),\\ FN(t)\;=\;\sum_{e_{1}\in \{1,\ldots ,n\}}\;(IoU*(O_{GT}(e_{1}),\;O_{E}(e_{2}))<t),\\ FP(t)\;=\;\sum_{e_{2}\in \{1,\ldots ,m\}}\begin{array}{r} \forall e_{2}\in \{1,\ldots ,m,\},\\ \;(IoU*(O_{GT}(e_{1}),\;O_{E}(e_{2}))<t),\\ \forall e_{1}\in \{1,\ldots ,n,\}, \end{array} \end{array}$$

and

$$\;()=\{\;\begin{array}{l} 1\;\text{if}\;\;\text{is}\;\text{true,}\\ 0\;\text{otherwise.} \end{array}$$

With a threshold *t* = 0.05, this metric gives the accuracy of a method to identify the correct number of nuclei, while with thresholds in the range 0.05 *−* 0.9, it evaluates the localization accuracy of the identified nuclear contours.

### Conditional GAN

The annotated 1868 × 1400 image was divided into thirty-five 256 × 256 images for training. As defined in 13, U-Net ^14^ was used for the generator and a convolutional PatchGAN classifier was used for the discriminator. Once trained, nuclei masks had to be generated to simulate images. Distributions for the number of nuclei per image and the size of nuclei were defined from the training dataset. The number of nuclei per image was then modeled as a Gaussian distribution while the size of nuclei was modeled by a Gumbel distribution to reflect the heavy tail distribution observed in the training dataset. Nuclei masks were then defined as ellipses randomly generated with these distributions with random orientation and a ratio between the two axes defined according to a Gaussian distribution of average *s/π* and standard deviation of 0.2 *s/π*, where *s* is the area of the ellipse. 1000 256 × 256 nuclei images were simulated by considering the generated ellipses as nuclei masks.

### Combination of instance and semantic segmentations

The combination of results obtained with instance and semantic segmentations was initialized as the nuclei segmented with Mask R-CNN. To prevent from hallucinations, nuclei identified with Mask R-CNN for which the area overlapping with nuclei obtained with U-Net was inferior to 20% were discarded. Then, nuclei identified with U-Net whose area overlapping with nuclei obtained with Mask R-CNN was inferior to 33% were added as new nuclei to the final segmentation. Finally, nuclei with an area inferior to 35 pixels were discarded.

## Results

### Deep learning-based instance segmentation with existing datasets and massive data augmentation is used to initialize the training dataset

**Figure 1.**
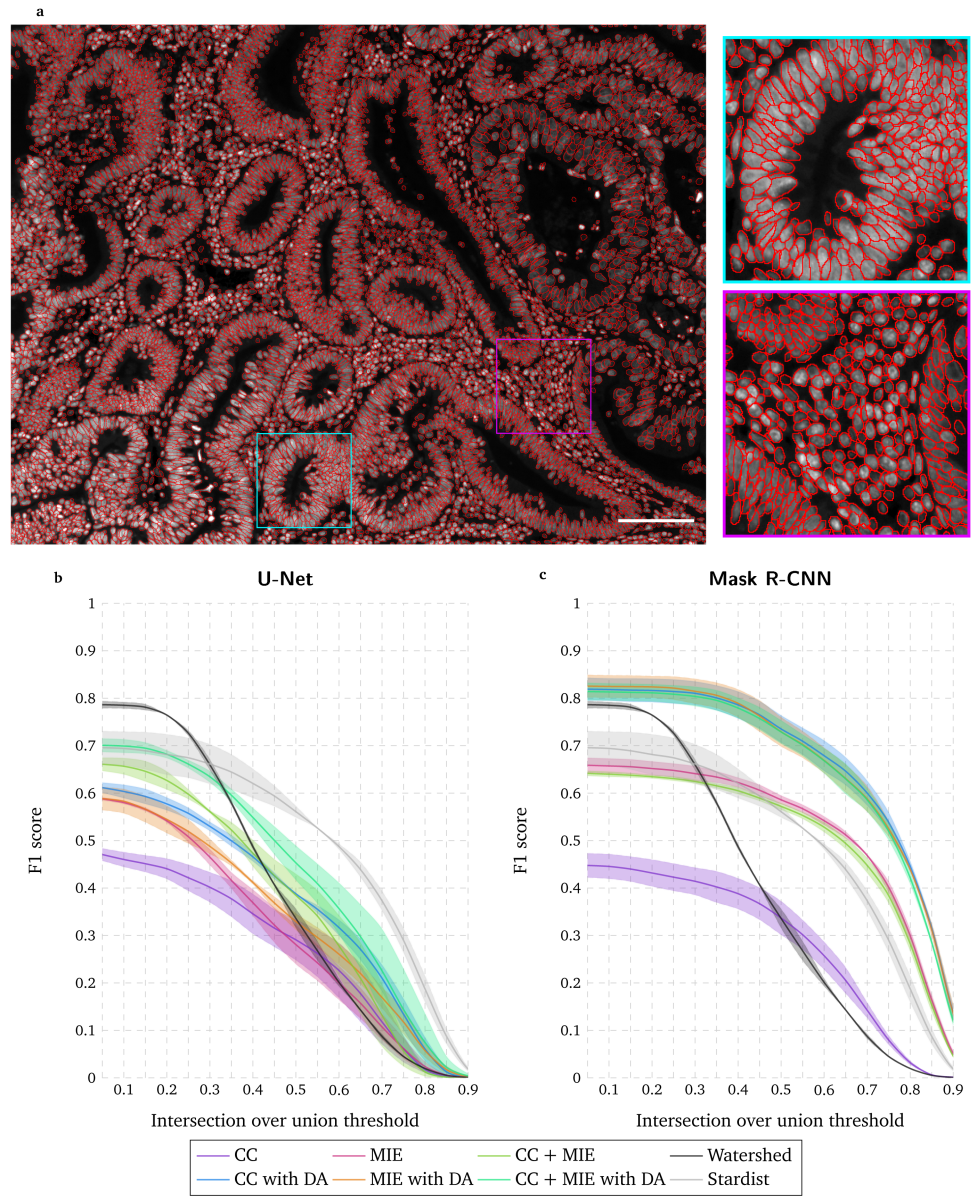
Manual annotation and evaluation of deep learning-based segmentation with existing training datasets. **a** Widefield acquisition of a human polyp biopsy stained with DAPI. Manually annotated nuclei are overlaid as red circles. Zoomed-in regions are displayed on the right side with corresponding squared colors. Scale bar = 100µm. **b**– **c** F1 score for range of IoU thresholds obtained with the watershed method, with Stardist, with U-Net **b** and Mask R-CNN **c** approaches trained with a high-throughput chemical screen on U2OS cells dataset (CC) or/and a widefield mouse intestinal epithelium dataset (MIE), with and without data augmentation (DA). Lines correspond to average F1 score over the two tested images while the shaded areas represent the standard deviation.

A training dataset is required to train a deep learning method for object segmentation. While new approaches emerge for this task such as interactive machine learning ^30^, users most often start with manually annotating objects of interest with existing annotation tools ^31, 32^. As shown in Figure 1a, this task is particularly challenging in our case due to the wide range of morphologies and high density of nuclei in polyps. We use the ImageJ plugin Annotater ^12^ to efficiently annotate nuclei, a task that takes approximately 30 hours for the image shown in Figure 1a. To avoid a fully manual annotation and save time, it is possible to use the same plugin to correct a nuclei segmentation obtained with an existing method. The watershed method ^33^, probably the most used method for nuclei segmentation in fluorescence microscopy images, correctly identifies a high number of nuclei (high F1 score for a low IoU threshold in Figure 1). Unfortunately, under- and over-segmentations, a well-known limitation of this approach, lead to a poor segmentation localization (rapidly decreasing F1 score with increasing IoU thresholds in Figure 1). Alternatively, pretrained deep learning models for nuclei segmentation are available. Stardist ^7^, one of the most popular approaches in microscopy, can be processed as a Fiji plugin ^25^ with a model trained on the 2018 Data Science Bowl ^10^. While the number of nuclei correctly identified is lower than with the watershed method (lower F1 score for low IoU thresholds in Figure 1b–c), their localization accuracy is much higher (higher F1 score for high IoU thresholds in Figure 1b–c). Another possibility is to train deep learning approaches with existing training datasets. We propose to train a U-Net model and a Mask R-CNN model with a high throughput chemical screen on U2OS cells dataset (CC) (image set BBBC039v1 available from the Broad Bioimage Benchmark Collection ^9^) and a widefield mouse intestinal epithelium dataset (MIE) ^12^. These models are then used to segment the image shown in Figure 1a. While U-Net demonstrates a poor performance ( Figure 1b), Mask RCNN clearly surpasses the watershed approach and the pretrained Stardist model ( Figure 1c). When compared to the latter, the good performance of Mask R-CNN is explained by the fact that the MIE dataset includes epithelial nuclei, even though they come from mice. Correcting this segmentation with Annotater takes about 15–20 hours, which is clearly faster than an annotation from scratch. Training the Mask R-CNN model with the CC and MIE datasets takes about 12 hours but has the great advantage that it does not require human interaction. For both UNet and Mask R-CNN, a massive data augmentation (100 times) clearly improves the performance.

**Figure 2.**
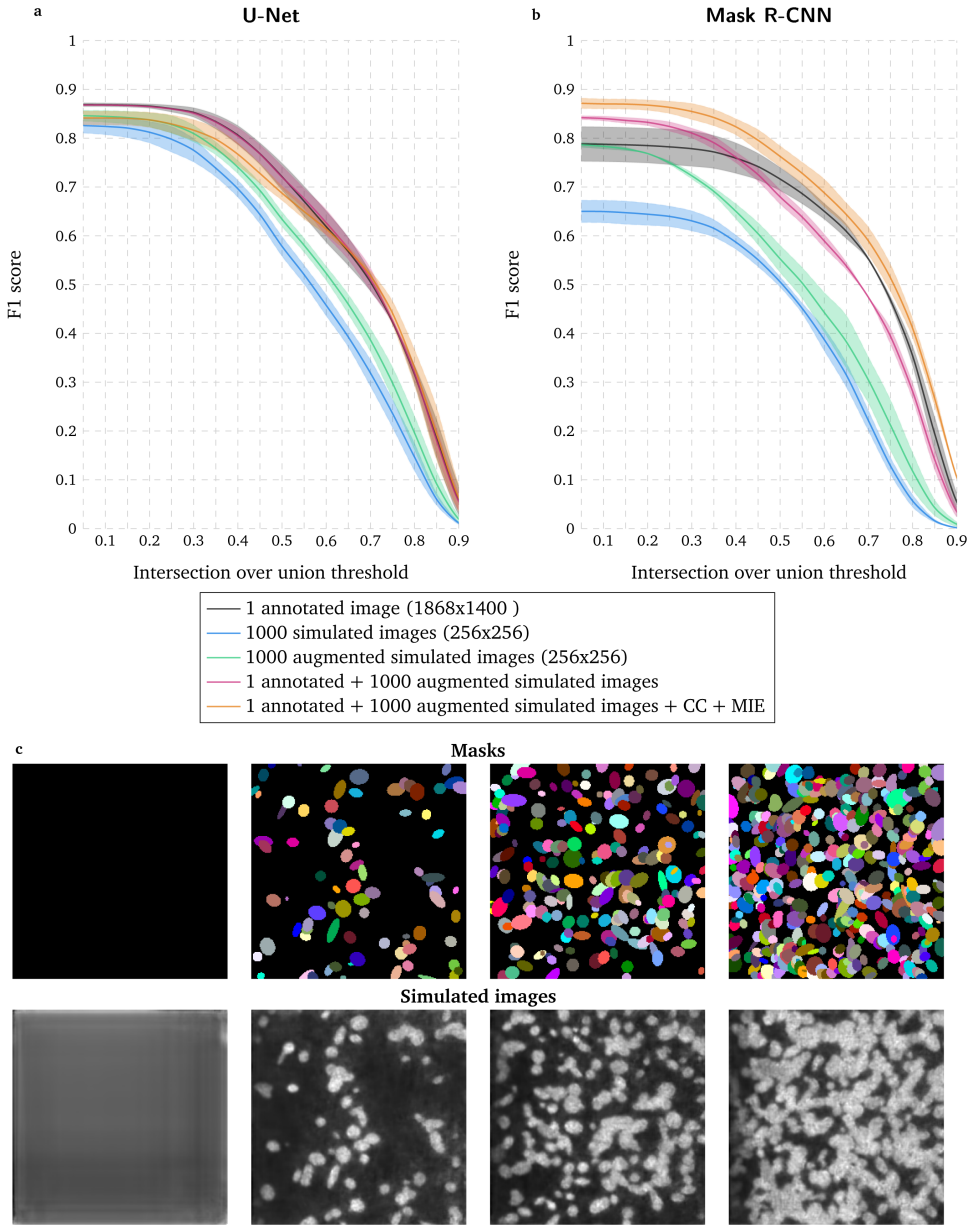
Evaluation of deep learning-based segmentation when using a conditional Generative Adversarial Network to increase the size of the training dataset. **a** First row: masks generated as ellipses (see Methods) and represented with unique colors. Second row: images simulated from masks shown in first row with a conditional Generative Adversarial Network (GAN). **b**– **c** F1 score for range of IoU thresholds obtained with U-Net **b** and Mask R-CNN **c** trained with 1 annotated image with data augmentation (DA), 1000 simulated images, 1000 augmented simulated images, 1 annotated image with DA combined with 1000 augmented simulated images and 1 annotated image with DA combined with 1000 augmented simulated images as well as a high-throughput chemical screen on U2OS cells dataset (CC) and a widefield mouse intestinal epithelium dataset (MIE). Lines correspond to average F1 score over the two tested images while the shaded areas represent the standard deviation.

**Figure 3.**
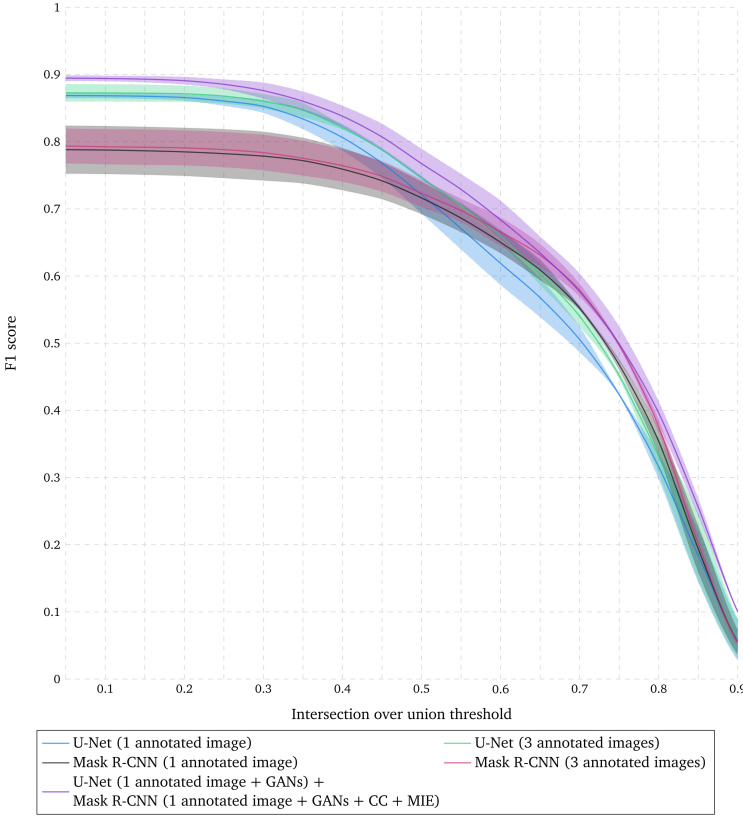
Evaluation of nuclear segmentation when combining U-Net and Mask R-CNN. F1 score for range of IoU thresholds obtained with U-Net trained with 1 and 3 annotated images with data augmentation (DA), Mask R-CNN trained with 1 and 3 annotated images with DA, and the combination of results obtained with U-Net trained with 1 annotated image with DA and augmented simulated images, and the results obtained with Mask R-CNN trained with 1 annotated image with DA, augmented simulated images and existing datasets with DA. Lines correspond to average F1 score over the two tested images while the shaded areas represent the standard deviation.

**Figure 4.**
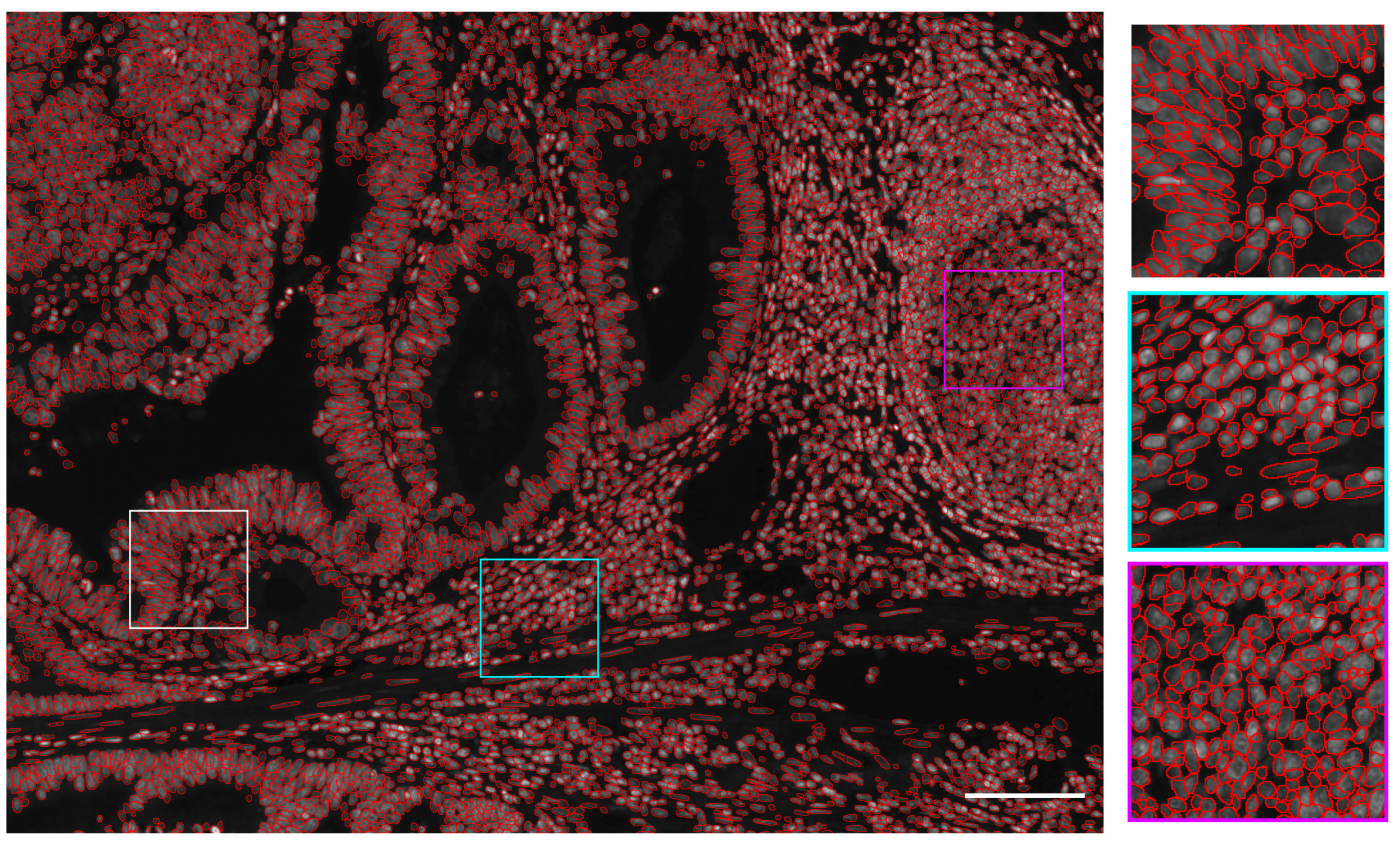
Nuclear segmentation example when combining U-Net and Mask R-CNN. Segmented nuclei obtained by combining U-Net and Mask R-CNN overlaid as red circles over the processed image. Zoomed-in regions are displayed on the right side with corresponding squared colors. Scale bar = 100µm.

### Increasing the training dataset by using a conditional GAN improves nuclear segmentation accuracy

When only considering the annotated image in Figure 1a in the training dataset, U-Net leads to higher segmentation accuracy than Mask R-CNN ( Figure 2a-b). To increase the training dataset, we use the same annotated image to train a conditional Generative Adversarial Network (GAN) ^13^ and simulate images showing nuclei from masks defined as random ellipses generated with the distributions of nuclei size and nuclei number observed in the training dataset (see Figure 2c and Methods). Only using simulated images lead to a lower accuracy for both deep learning approaches, even though applying mathematical operations to these synthetic images (augmented simulated training dataset, see Methods) improves the segmentation accuracy. However, pooling together augmented simulated images and the annotated image from Figure 1a slightly improves U-Net performance and distinctly increases the number of accurately identified nuclei with Mask R-CNN while decreasing the segmentation localization precision. Finally, adding existing datasets clearly leads to the optimal results for Mask R-CNN while degrading the accuracy for U-Net, which is consistent with the inability for this approach to generalize nuclear segmentation for different data as shown in Figure 1b. Overall, U-Net marginally benefits from using simulated images (red curve versus black curve in Figure 1a) while the main gain for Mask R-CNN comes from the use of CC/MIE datasets and data augmentation (orange curve versus red and black curves in Figure 1b).

### Combining semantic and instance segmentations improves nuclear segmentation accuracy

Nuclei segmented with Mask R-CNN show a higher localization precision than those obtained with U-Net as shown in Figure 1a-b. However, nuclei that are harder to delineate are missed with Mask R-CNN while U-Net accurately identifies pixels that belong to nuclei, even though the separation between individual nuclei might not be precise. In order to get the best of both worlds, we propose to combine the results obtained with U-Net trained with one annotated image with data augmentation and augmented simulated images, and the results obtained with Mask R-CNN trained with one annotated image with data augmentation, augmented simulated images and existing datasets with data augmentation (see Methods). As shown in Figure 3, these results demonstrate a higher F1 score for any IoU threshold than obtained with U-Net or Mask R-CNN trained with 3 times more annotated images. The corresponding segmented nuclei are shown in Figure 4.

## Discussion

This study explores several strategies to minimize the amount of manually annotated data required to successfully train a deep learning model for instance segmentation. As already established in the field, the use of existing training datasets, even though modalities and/or tissues differ, allows to train instance segmentation models and obtain results on targeted data that can be manually corrected to initialize a new training dataset. The use of massive data augmentation is another well known approach to drastically increase the segmentation accuracy. While the use of conditional GANs to expand the size of the training dataset seems promising, the gain in accuracy shown in this study is not very convincing. The simulation pipeline used to generate the masks might have been too simplistic. In particular, the variety of nuclei shapes could be enriched. Finally, combining semantic and instance segmentation results leads to a substantial increase in segmentation accuracy. While unusual in the field, we believe that this method has the potential to become more common in the community. Combining these strategies enables to remarkably reduce the amount of data to be manually annotated, waiting for methods that offer the promise to eliminate this time consuming task, such as self- and partially supervised methods, currently in development.

## Data availability

The five annotated images are available at https://github.com/tpecot/DeepLearningBasedSegmentationForBiologists/tree/main/Data/AnnotatedNuclei. This project contains the following data:

- *Polyp12_[10837,39273]_component_data.tiff*: image used for training U-Net and Mask R-CNN in all figures and for training pix2pix in Figure 2
- *Polyp40_[13694,34105]_component_data.tiff and Polyp42_[12011,37598]_component_data.tiff*: two images used for training U-Net and Mask R-CNN in Figure 3
- *Polyp12_[12699,39273]_component_data.tiff and Polyp42_[12942,36900]_component_data.tiff*: two images used for evaluation in all figures

The images generated with pix2pix and used for training U-Net and Mask R-CNN in Figure 2– Figure 3 are available at https://github.com/tpecot/NucleiSimulationWithConditionalGAN/tree/main/datasets/Nuclei_polyps_1image.

## Software availability

The code with the parameters used to train and process all experiments presented in this manuscript with U-Net and Mask R-CNN is available at https://github.com/tpecot/DeepLearningBasedSegmentationForBiologists/tree/main/Codes.

Archived code as at time of publication: https://doi.org/10.5281/zenodo.4608795 ^34^

License: GPL3

The code with the parameters used to train and generate images with pix2pix is available at https://github.com/tpecot/NucleiSimulationWithConditionalGAN.

Archived code as at time of publication: https://doi.org/10.5281/zenodo.4608793 ^35^

License: GPL3

The ImageJ macro used to convert the output classes obtained with U-Net to individual nuclei is available at https://github.com/tpecot/DeepLearningBasedSegmentationForBiologists/tree/main/Codes.

Archived macro as at time of publication: https://doi.org/10.5281/zenodo.4608795 ^34^

License: GPL3
