# Supplementary material for: A deep learning segmentation strategy that minimizes the amount of manually annotated images
Source: F1000Res. 2022 Jan 17;10:256. Originally published 2021 Mar 30. [Version 2] doi: 10.12688/f1000research.52026.2 (PMC8787559; doi:10.12688/f1000research.52026.2)
Supplement: Peer review history

# Peer review: Reviewer response for version 2

**DOI:** 10.5256/f1000research.115746.r120021

**Published:** 2022-01-24

**Authors:** Romain F Laine

**Affiliations:** 1 MRC Laboratory for Molecular Cell Biology, University College London, London, WC1E 6BT, UK

The authors have now fully addressed my comments. This manuscript makes a nice and timely story, I have enjoyed reading and helping reviewing it. Nice work!

Is the rationale for developing the new method (or application) clearly explained?

Yes

Is the description of the method technically sound?

Yes

Are the conclusions about the method and its performance adequately supported by the findings presented in the article?

Partly

If any results are presented, are all the source data underlying the results available to ensure full reproducibility?

Yes

Are sufficient details provided to allow replication of the method development and its use by others?

Yes

Reviewer Expertise:

I am a quantitative imaging specialist, focused on fluorescence microscopy, super-resolution, and quantitative analysis method development, including deep learning.

I confirm that I have read this submission and believe that I have an appropriate level of expertise to confirm that it is of an acceptable scientific standard.

# Peer review: Reviewer response for version 2

**DOI:** 10.5256/f1000research.115746.r120022

**Published:** 2022-01-24

**Authors:** Alice Lucas

**Affiliations:** 1 Broad Institute, Cambridge, MA, USA

Thank you for addressing my earlier comments.

Is the rationale for developing the new method (or application) clearly explained?

Yes

Is the description of the method technically sound?

Yes

Are the conclusions about the method and its performance adequately supported by the findings presented in the article?

Yes

If any results are presented, are all the source data underlying the results available to ensure full reproducibility?

Yes

Are sufficient details provided to allow replication of the method development and its use by others?

Yes

Reviewer Expertise:

Deep Learning, Computer Vision, Image and Video Processing

I confirm that I have read this submission and believe that I have an appropriate level of expertise to confirm that it is of an acceptable scientific standard.

# Peer review: Reviewer response for version 1

**DOI:** 10.5256/f1000research.55252.r101244

**Published:** 2021-12-14

**Authors:** Romain F Laine

**Affiliations:** 1 MRC Laboratory for Molecular Cell Biology, University College London, London, WC1E 6BT, UK

Pecot *et al* present a nice set of ideas about how to improve the pipeline of nuclei segmentation. The premise of this work is that it is time consuming to generate good quality annotation for DL training. The authors are absolutely right here, it takes time and can be discouraging.

 The authors test a couple of interesting approaches to help with that:

1. Use of large openly accessible dataset to create pretrained dataset, as is commonly done in the field.
2. Use of data augmentation to improve generalization of the model, also commonly done in the field already.
3. The use of a generator model (here pix2pix as a conditional GAN), to expand the size f the training dataset.
4. Combine output of 2 common segmentation networks (U-Net and MaskRCNN) to improve accuracy.

The points 1 and 2 are already well established in the field and will be systematically done nowadays, with almost any DL networks when data is available. Segmentation dataset are available as the authors highlight. So these aspects are sanity checks here and not novel implementations. However, it is reassuring to see that augmentation and use of pretrained models are helpful here as well.

 The more interesting aspects of this work lie in the use of GAN for expanding the size of the training dataset from an annotated image and the combination of output. Although the use of GAN makes sense for this application, the gains from such approach are clearly quite marginal as can be seen on Figure 2a and 2b comparing the black and red lines, while the main gains are again from the use of additional dataset and augmentation as observed on Figure 1. It's an important observation but maybe not as essential to the pipeline as is described in the manuscript as it stands. I suggest toning down the importance of this and clearly highlighting that the gains are in fact low here. 

 Maybe the authors could further discuss why the gains are only small here: maybe the simulation pipeline from the masks to generate the training dataset of pix2pix is too simplistic for instance, wider range of shapes, background lights, heterogeneity of intensities or patterns on the nuclei etc.

 On the contrary it's quite clear that the combination of U-Net and MaskRCNN output are beneficial to the overall performance of the method and that's nicely shown here. I think that combining DL model outputs is currently underused and this is a nice additional demonstration of this here.

 As additional comments, I would highlight a couple of additional work that are missing from the context described in this paper:

- Kaibu (Wei Ouyang *et al *F1000, https://f1000research.com/articles/10-142) is an interactive tool for simultaneous training of segmentation models and segmentation, this circumvents a range of issues mentioned here, it should be mentioned.
- StarDist (https://github.com/stardist/stardist) from Uwe Schmidt and Martin Weigert, is an excellent tool for nuclei segmentation and is not included here. I suggest that the authors compare their IoU curves to those obtained from the pre-trained models provided by the method (even as Fiji plugin). This will give the readers a baseline on which to compare the approaches described here, which still require an investment in time to train multiple models and annotations
- The cost/benefit analysis of manual annotation vs automated (DL based or not) should be mentioned, it's not always worth doing DL for that, it often depends on the size of the dataset to be segmented.
- Although having an annotator GUI and some tools to get some improvement on segmentation performance are important today, a large efforts is now put into approaches that are self-supervised or partially supervised, which would circumvent the issues of annotation time altogether. These are not currently available to the wide bioimaging community but should be mentioned in conclusion, looking at the future of segmentation pipelines.

Overall, I think that it is a nice piece of work describing the performance of a range of approaches in a systematic and clear manner, which are useful to the bioimaging community. However, they are presented as guidelines to building a segmentation pipeline and I would not think that as such, it describes the general thoughts about the matter in the community. I'd consider rewording the conclusions focusing on the observations of the tests the authors made rather than presenting it as a universal guideline.

Is the rationale for developing the new method (or application) clearly explained?

Yes

Is the description of the method technically sound?

Yes

Are the conclusions about the method and its performance adequately supported by the findings presented in the article?

Partly

If any results are presented, are all the source data underlying the results available to ensure full reproducibility?

Yes

Are sufficient details provided to allow replication of the method development and its use by others?

Yes

Reviewer Expertise:

I am a quantitative imaging specialist, focused on fluorescence microscopy, super-resolution, and quantitative analysis method development, including deep learning.

I confirm that I have read this submission and believe that I have an appropriate level of expertise to confirm that it is of an acceptable scientific standard, however I have significant reservations, as outlined above.

## References

[1] : Interactive biomedical segmentation tool powered by deep learning and ImJoy. *F1000Research* .2021;10: 10.12688/f1000research.50798.1 10.12688/f1000research.50798.1

# Response

**Published:** 2021-12-17

**Authors:** Thierry Pecot

**Affiliations:** University of Rennes 1, France

We thank Romain Laine for his enlightening observations.

 To answer Dr Laine remarks, we changed the manuscript accordingly:

- We added a sentence about the cost/benefit analysis of manual annotation vs automated in the introduction.
- We mentioned the use of interactive machine learning and cited Kaibu (Wei Ouyang *et al *F1000, https://f1000research.com/articles/10-142) at the beginning of the first section.
- We added a comparison to Stardist trained with the 2018 Science Bowl (Fiji plugin) in Fig.1. We then compared its performance to the watershed approach, to U-Net and to Mask R-CNN trained with the CC/MIE datasets in the first section.
- We completely changed the discussion, focusing on the observations made in the manuscript. More particularly, we acknowledge that the use of publicly available datasets and massive data augmentation are beneficial to build a training dataset and are now common practices in the field. We also underline the disappointing accuracy obtained when using pix2pix (we also changed the end of section 2 accordingly). We emphasize the interest of combining instance and semantic segmentations. We finally introduce self- and partially supervised methods that offer the promise to eliminate manual annotation.

# Peer review: Reviewer response for version 1

**DOI:** 10.5256/f1000research.55252.r89422

**Published:** 2021-08-03

**Authors:** Alice Lucas

**Affiliations:** 1 Broad Institute, Cambridge, MA, USA

The authors propose multiple strategies to improve segmentation results given a new dataset.

 Instead of manually annotating a training image from scratch, the authors recommend to leverage knowledge learned by networks pre-trained on other larger datasets. Therefore, they propose to first train a model on large existing datasets (that differ from the final dataset of interest). The trained model is then used to annotate the desired training image, and these predictions are then manually corrected using Annotater. This allows the authors to manually annotate for 15-20 hours, compared with 30 hours when annotating the image from scratch.

 A second solution that they implement in order to improve their final segmentation results is to (1) train a conditional GAN on their annotated image and (2) use the cGAN to predict additional synthesized segmentation masks. The UNet and Mask-RCNN can then use this additional data for training.

 Finally, to further improve their results, they combine results obtained from their trained UNet and their trained Mask-RCNN to obtain a final instance segmentation map. More specifically, the semantic segmentations from UNet are post-processed to obtain instance segmentation masks, and merged (following a specific protocol) with those predicted by the trained Mask-RCNN.

 A few comments: 

- Clarity regarding the purposes of the different training sets used could be improved. At first it was not clear to me how the CC/MIE datasets related to the final training dataset of interest (the 1868 x 1400 image). It could be made a bit more explicit that (1) the CC / MIE data is used to pre-train a neural network, (2) this neural network is then applied on the image of interest to provide the annotations, and (3) final training data is obtained by correcting these predictions. (4) On this final training data will be trained UNet and Mask-RCNN.
- The text “Only one image was used to train […]” is a bit of a misleading statement. In the end, when looking at the whole pipeline, a very large dataset of annotated images was used to get to these results.
- It would be interesting to know how many hours it took to pre-train Mask-RCNN and UNet on the large datasets, as well as for training the conditional GAN. This is helpful especially for better comparing the 30 hours of manual annotation from scratch vs. the 15-20 hours when using these strategies.

Is the rationale for developing the new method (or application) clearly explained?

Yes

Is the description of the method technically sound?

Yes

Are the conclusions about the method and its performance adequately supported by the findings presented in the article?

Yes

If any results are presented, are all the source data underlying the results available to ensure full reproducibility?

Yes

Are sufficient details provided to allow replication of the method development and its use by others?

Yes

Reviewer Expertise:

Deep Learning, Computer Vision, Image and Video Processing

I confirm that I have read this submission and believe that I have an appropriate level of expertise to confirm that it is of an acceptable scientific standard, however I have significant reservations, as outlined above.

# Response

**Published:** 2021-12-17

**Authors:** Thierry Pecot

**Affiliations:** University of Rennes 1, France

We thank Alice Lucas for her insightful remarks and apologize for the delay between her review and our response, we were waiting for a second reviewer.

 To answer Dr Lucas comments, we changed the manuscript accordingly:

- We rephrased the first section to better explain what was done. More specifically, U-Net and Mask R-CNN are trained with CC/MIE datasets along with a massive data augmentation. The trained models are then used to segment the image shown in Fig.1 **a**. The accuracy obtained with these models is compared to the watershed approach and to a Stardist model trained with the 2018 Data Science Bowl. As Mask R-CNN demonstrates the most accurate results, the segmented nuclei obtained with this approach are then manually corrected to initialize a training dataset.
- To clarify the misleading text “Only one image was used to train […]” , we changed the **Training dataset** section in **Methods** and added that publicly available datasets were used in addition to the manually annotated image of human precancerous polyp biopsy.
- We added a sentence about the time taken to train a Mask R-CNN model on the CC/MIE datasets at the end of the first section to better compare the 30 hours of manual annotation from scratch vs. the 15-20 hours when using this strategy.
